# Generation of crystal structures using known crystal structures as analogues

**DOI:** 10.1107/S2052520616006533

**Published:** 2016-07-16

**Authors:** Jason C. Cole, Colin R. Groom, Murray G. Read, Ilenia Giangreco, Patrick McCabe, Anthony M. Reilly, Gregory P. Shields

**Affiliations:** a Cambridge Crystallographic Data Centre, 12 Union Road, Cambridge, Cambridgeshire CB2 1EZ, England

**Keywords:** crystal structure prediction, shape searching, Cambridge Structural Database

## Abstract

This analysis attempts to answer the question of whether similar molecules crystallize in a similar manner. An analysis of structures in the Cambridge Structural Database shows that the answer is yes – sometimes they do, particularly for single-component structures. However, one does need to define what we mean by *similar* in both cases. Building on this observation we then demonstrate how this correlation between shape similarity and packing similarity can be used to generate potential lattices for molecules with no known crystal structure. Simple intermolecular interaction potentials can be used to minimize these potential lattices. Finally we discuss the many limitations of this approach.

## Abstract 2

An investigation into using shape-similarity of molecules to generate putative crystal structures.

## 1. Introduction   

Effective and efficient methods for crystal structure prediction (CSP) are much sought after (Price, 2014 ▸). Much of the focus of these methods has been directed towards identifying the thermodynamically most stable lattice a molecule can form. This is a formidable challenge. Firstly, one must identify plausible conformations of the molecule of interest. An enormous range of potential lattices consisting of these conformers must then be generated. These trial structures are then typically subjected to energy minimization and the resulting energies calculated to identify those with the lowest.

This typical procedure requires significant expertise and carries with it a high computational cost, so CSP tends only to be applied in cases of high worth. Recent CSP studies include examples of pharmaceuticals, *e.g.* axitinib (Vasileiadis *et al.*, 2015 ▸), fenamates (Uzoh *et al.*, 2015 ▸; López-Mejías & Matzger, 2015 ▸), molecular electronic materials (Goldstein *et al.*, 2015 ▸) and energetic materials (Mendoza-Cortes *et al.*, 2016 ▸).

Our work is aimed at reducing the number of potential lattices one needs to generate and further reducing the number that must be subject to computationally expensive energy calculations to identify the potential structures with the lowest energy. A particular focus is the rapid generation of a relatively small number of potential crystal structures, containing a high proportion of those with the lowest energy – a so-called structural landscape.

Recent research has highlighted the importance of structural landscapes (*i.e.* a collection of putative structures: Price & Reutzel-Edens, 2016 ▸) in understanding polymorphism (Price, 2013 ▸; Cruz-Cabeza *et al.*, 2015 ▸), molecular conformation (Cruz-Cabeza & Bernstein, 2014 ▸; Thompson & Day, 2014 ▸) and co-crystallization (*e.g.* Chan *et al.*, 2013 ▸; Bučar *et al.*, 2013 ▸; Hoxha *et al.*, 2015 ▸). A useful structural landscape informs the researcher as to the likelihood or prospect of experimentally finding further, as-yet unobserved, forms. Existing methods for landscape generation typically utilize a global search algorithm to sample structural space. A force field is then used for structural ranking; this can either be a general force field (*e.g.* Kim *et al.*, 2009 ▸; Lund *et al.*, 2013 ▸) or a force field that has been tailored to the problem in hand (Neumann *et al.*, 2008 ▸; Kendrick *et al.*, 2011 ▸).

We set out to determine whether landscape generation could be made more effective and efficient by using more than 800 000 organic and organometallic crystal structures in the Cambridge Structural Database (CSD: Groom *et al.*, 2016 ▸) as a library of potential molecular packing arrangements. Indeed common packing arrangements in the CSD have been classified, *e.g.* the box model (Pidcock & Motherwell, 2004*a* ▸,*b* ▸; Pidcock, 2006 ▸), hexagonal packing classifiers (Braun & Huttner, 2005 ▸), and crystal packing similarity calculated using moments of inertia (Galek, 2011 ▸).

The CSD does contain many examples of isostructurality – where related molecular compounds crystallize with related packing arrangements (Kálmán *et al.*, 1993 ▸; Kálmán & Fábián, 2007 ▸). However, previous studies (*e.g.* Edwards *et al.*, 2001 ▸) have also shown that very small changes to the chemical structure can also induce very large changes to the packing arrangements. Despite this, the CSD can be seen as a database of possible packing arrangements. Indeed, Kitaigorodskii, in his seminal work of 1955, recognized the importance of molecular shape in defining crystal structures (Kitaigorodskii, 1961 ▸: English translation).

If we make the unqualified assertion that the CSD represents a set of the most common crystal packing arrangements, we should be able to use the CSD as a source of prior information to bias searching for likely crystal packing arrangements of related molecules in the context of structure prediction, by using the known crystal structures as packing analogues for molecules with unknown crystal structures. This has the potential to focus searches in more frequently populated regions of the high-dimensional search space and so improve performance. The challenges of such an approach are not inconsiderable however; how do we choose the correct structures to use as possible analogues? This paper deals with the problem of analogue selection and attempts to determine whether the CSD is indeed representative of common structural packings.

## 2. Materials and methods   

### 2.1. Definition of terms   

Various terms are used to describe the nature of crystal structures. We follow the definitions given by Steed & Steed (2015 ▸). Namely:

*Z*′ is defined as the number of formula units in the asymmetric unit of the unit cell.

*Z*′′ is defined as the number of discrete molecules in the asymmetric unit of the unit cell.

*Z* ^r^ is defined as the number of distinct types of molecule (chemical residues) in the asymmetric unit.

### 2.2. Shape analogue methodology   

#### 2.2.1. Overview   

We attempt to find existing crystal structures that could be packing analogues for an unknown crystal structure, comprising one or more target molecules. More precisely, we attempt to do this for a single conformer of a target molecule, although the method could be extended by multiple repetitions with probable conformers generated by a suitable conformer generation algorithm (*e.g.* Vainio & Johnson, 2007 ▸; Hawkins *et al.*, 2010 ▸; Miteva *et al.*, 2010 ▸; O’Boyle *et al.*, 2011 ▸; Korb *et al.*, 2014 ▸). Here we evaluate the simplest possible scenario; we use the conformation in the observed crystal structure for shape searching of our test molecules, and evaluate the performance of our method as an information-driven approach for structural landscape generation.

**Figure 1:**
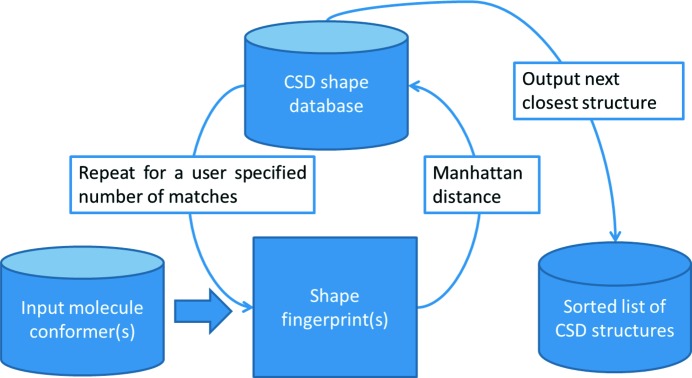
Shape match method against the CSD.

**Figure 2:**
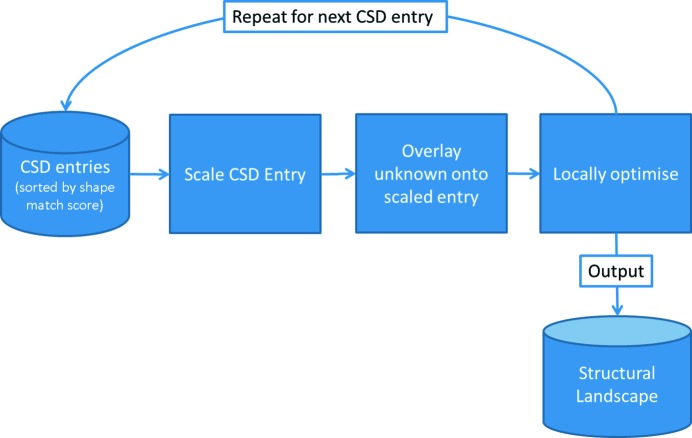
Overlay method for CSD entries sorted by shape match score.

Our method comprises two separate steps. In step 1 (Fig. 1 ▸), a molecular shape fingerprint database, derived from the CSD, is searched to retrieve a set of CSD entries sorted by shape match order. Each hit CSD structure is scaled isotropically, so that the total volume of the hit CSD molecule(s) matches the total volume of the target molecule(s), to generate an analogue structure. In the analogue structure, the geometry of the molecule(s) may not be chemically reasonable due to the scaling, but they represent the desired molecular shape(s). Subsequently, each target molecule is aligned with its corresponding shape-matched molecule in the analogue structure. In step 2 (Fig. 2 ▸), the resultant crystal structure is locally optimized.

#### 2.2.2. Shape matching in the CSD   

Shape fingerprints are calculated using ultrafast shape recognition (USR; Ballester & Richards, 2007 ▸) for each molecule in the CSD. Each CSD structure is then associated with one or more fingerprints (one fingerprint per separate molecular entity in the CSD entry).

For supplied molecular conformation(s) of the target molecule(s), a query shape fingerprint is calculated. This fingerprint is then used to search the database of molecular shapes in the CSD. Shape fingerprint matches are scored based on their Manhattan distance (Krause, 1988 ▸) from the query to the respective molecule in the CSD entry. This shape match score is converted to a molecule match score by taking the negative log of the probability of encountering a higher shape match score in the CSD. These log probabilities are used so as to allow molecule match probabilities in multi-component systems to be combined additively. The log probabilities are interpolated from a pre-calculated table derived from shape match score frequencies observed in the CSD (see §S1 of the supporting information).

All CSD structures are ranked according to their molecule match scores. For prediction of multi-component structures, all molecular components are shape-searched against CSD structures that contain the same number of components (*Z* ^r^) as the target system. For each CSD structure, the combination that gives the highest total sum of molecule match scores is found. This total sum is used as the structure’s score. The CSD structures returned as analogues are those that have the highest structure scores.

#### 2.2.3. Structural alignment   

While shape-matching provides possible CSD entries as start points, the problem of alignment into the underlying lattice still has to be solved. To achieve this, alignment of molecules is performed using the CCDC ligand overlay tool (Taylor *et al.*, 2012 ▸).

Our method is parameterized to perform volume-only alignment(s) of the target molecule(s) with the observed location(s) of their respective shape-matched molecule in the associated structure.

The overlay protocol used is not always successful, particularly with highly symmetrical, very small or very dissimilar molecules. For such systems, we place the target molecule(s) into the unit cell such that their centroids are overlaid with their respective shape-matched molecules. To prevent unwanted bias in our evaluation, the input structures are first randomly oriented in space, so any pre-alignment of the input coordinates with the observed structure is eliminated.

### 2.3. Structure model and scoring   

Our structural model is composed of cell lengths and angles (6 variables), molecule position and orientation (6 variables per independent molecule). Cell angles are fixed if constrained by space-group symmetry; otherwise no restrictions are placed on the allowed range of variables in local optimization. Given the molecules that form the asymmetric unit, initial cell variables and a fixed space group, a central unit cell is constructed from the asymmetric unit by applying the space-group symmetry operators. Surrounding unit cells are added by a breadth-first search of neighbouring unit cells until there are no more unit cells that contain atoms within 12 Å of the asymmetric unit atoms. During local optimization, if more unit cells are found to contain atoms within 12 Å of the asymmetric unit atoms, they are also included and the optimization restarted from its current position.

An intermolecular score *S(inter)* for a crystal structure is calculated on a per-molecule basis using intermolecular atom–atom Buckingham potentials. The potential parameters are based on the Unimol intermolecular force-field described by Filippini & Gavezzotti (1993 ▸) and Gavezzotti & Filippini (1994 ▸), tuned to improve their ability to replicate CSD crystal structures (see §S2 of the supporting information for details).

Local gradient-based minimization of the total score *S*(total) is carried out using an implementation of the Limited Memory BFGS method (Liu & Nocedal, 1989 ▸). For implementation efficiency, gradients are calculated using the CppAD automatic differentiation library (Bell, 2015 ▸).

### 2.4. Test set   

Initially, 379 structures of drug molecules, identified from DrugBank (Wishart *et al.*, 2006 ▸) and present in the CSD, were added to a test set of structures for prediction. Where more than one entry was available for a given molecule, the most recent entry with coordinates deposited in the CSD was selected for inclusion. This selection procedure broadens the range of space groups observed in the test set as there is a tendency for later entries in CSD entry families to have less common space groups (see §S3 of the supporting information for a more detailed analysis of this phenomenon). 19 entries were then excluded due to disorder in the observed structure, having *Z*′ < 1, having improbable bond lengths or angles (Bruno *et al.*, 2004 ▸), or having a duplicate already in the list.

**Figure 3:**
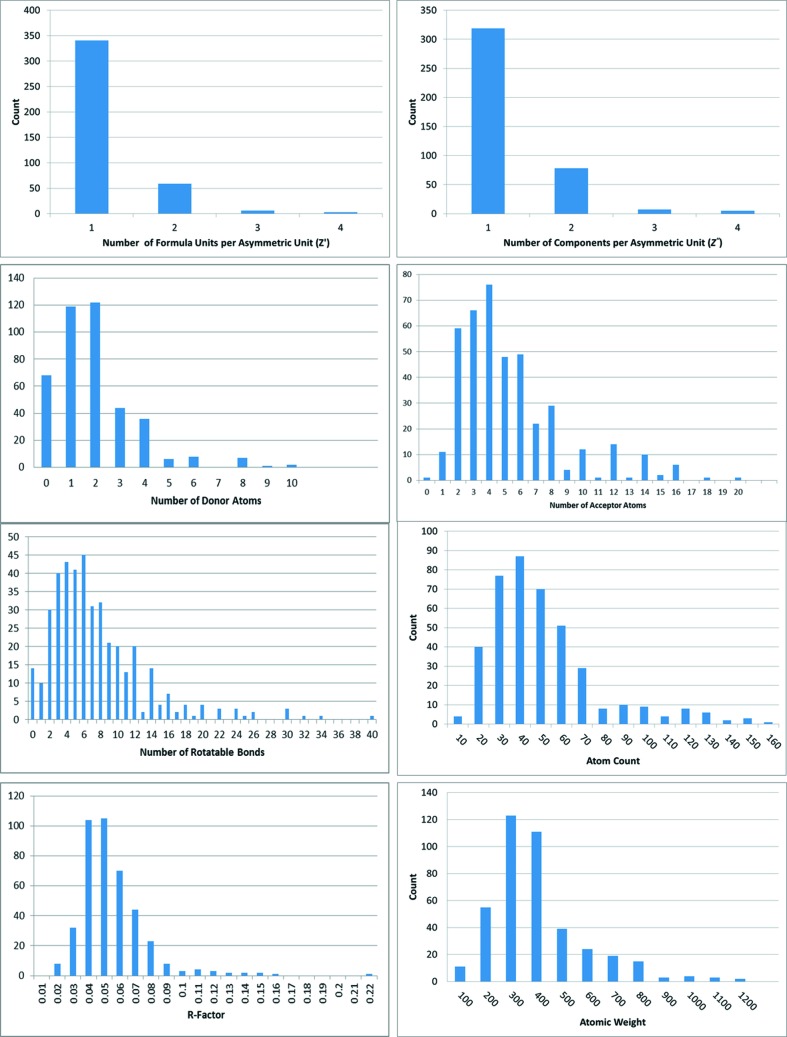
Distributions of summary descriptors of the 409 test set entries.

The test set was augmented with the 18 single-component *Z*′ = 1 structures used in the first five previous blind tests of crystal structure prediction methods (Lommerse *et al.*, 2000 ▸; Motherwell *et al.*, 2002 ▸; Day *et al.*, 2005 ▸, 2009 ▸; Bardwell *et al.*, 2011 ▸), another 30 structures used in previous studies of crystal minimization (Karamertzanis & Price, 2006 ▸; Kim *et al.*, 2009 ▸), and alphabetically the first structure in the CSD, reference code AABHTZ. The final set of 409 structures represents a broad selection of drug-like crystal structures in the CSD and we would encourage reuse in other evaluations of CSP methodology. Summary statistics and descriptor distributions including hydrogen-bond donor and acceptor counts are given in Table 1 ▸ and Fig. 3 ▸. The maximum *R*-factor in Table 1 ▸ is perhaps surprisingly high; however, the corresponding structure was determined using high-resolution powder diffraction.

### 2.5. Evaluation of the method   

We evaluated our methodology by comparison of predicted structures with minimized observed structures. The observed structures were locally minimized with our intermolecular force-field (the observed molecular conformation was fixed during minimization). This allowed us to evaluate the ability of the search methodology, independent of the performance of the scoring methodology. We define success for a given test set entry if we retrieve the structure that is found in the local minimum closest to the observed structure within the score’s response surface, rather than the observed structure itself. Structures were considered to be equivalent if the crystal packing similarity (Chisholm & Motherwell, 2005 ▸) of a cluster size of 15 molecules gave a (heavy-atom) RMSD (root mean-square deviation) less than 0.5 Å, with a distance tolerance of 20%, an angle tolerance of 20°.

365 structures in the test set minimize to a local minimum that has 15 molecules in common at an RMSD of 0.5 Å or less with the observed crystal structure, 388 have a cluster of 15 molecules in common at an RMSD of less than 1.0 Å. 15 structures had cluster sizes between 8 and 14 with an RMSD of less than 1.0 Å. The remaining 6 structures had smaller cluster sizes or had larger cluster sizes, but with RMSDs beyond 2.0 Å and can be regarded as failures within the underlying force field. A high proportion (15 out of 21) of the structures that have a cluster size less than 15 are multi-component (*Z*′ > 1), reflecting the higher complexity of these structures.

The shape database was searched with each unminimized test set molecule to retrieve the 100 000 best potential structural analogues for that molecule. CSD structures of the test set molecules themselves and their enantiomers were excluded, but no attempt was made to exclude structures of highly chemically similar molecules. Three alternative protocols were applied to the test set:

(i) ‘Full alignment’: The structures were searched in shape match order and the target molecules were structurally aligned with the molecules in the shape analogue.
(ii) ‘Centroid alignment’: The structures were searched in shape match order and the target molecule centroids were superimposed with the centroids of the molecules in the structural analogue, but the target molecules were placed in a random orientation within the structural analogue.
(iii) ‘Random’: Entries in the CSD were selected at random, but the target molecules were fully structurally aligned with the molecules in the structural analogue.

## 3. Results and discussion   

### 3.1. Overall performance   

**Figure 4:**
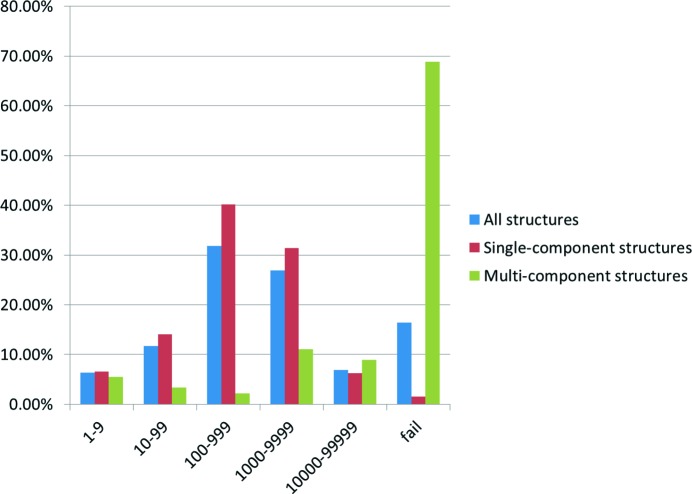
Percentage of test set entries that are retrieved within *N* structural analogues for all structures (*Z*′′ ≥ 1), single-component structures (*Z*′′ = 1) and multi-component (*Z*′′ > 1) structures is given.

Figs. 4 ▸, 5 ▸ and Table 2 ▸ show that for 83.6% of test set molecules an analogue structure in the CSD can be found that acts as a successful starting point for minimization to the same minimum as the observed crystal structure within 100 000 shape-matched CSD structures. This may seem like a large number, but actually represents a large reduction in the number of potential start points that are evaluated in many methods. The success rate in single-component structures is 98%. The success rate in multi-component structures is 31% despite the difficulties of multiple shape matching.

Taken at face value, this result would appear quite remarkable; one could interpret this as meaning very close representative ‘packings’ exist for almost all single-component structures of drug-like molecules and that the analogue-based search gives near complete coverage of the search space, but one needs to take care in interpretation. It could indicate that simply given enough random trial start points one increases the chance of finding the correct minimum to almost complete success. We therefore used a test set of molecules to establish the decrease in recovery of the observed crystal structure *via* local optimization with increasing degrees of random perturbation away from the observed structure. A complete description of the approach used is given in §S4 of the supporting information. The results show that with perturbations of up to 20% of all of the free variables in our model, one would expect a recovery rate of approximately 40%. Perturbations of up to 50% show a significant fall in recovery rate; only approximately 3% of optimizations result in the observed crystal structure, with a further long-tailed decline to 1.7% for 95% perturbation levels.

**Figure 6:**
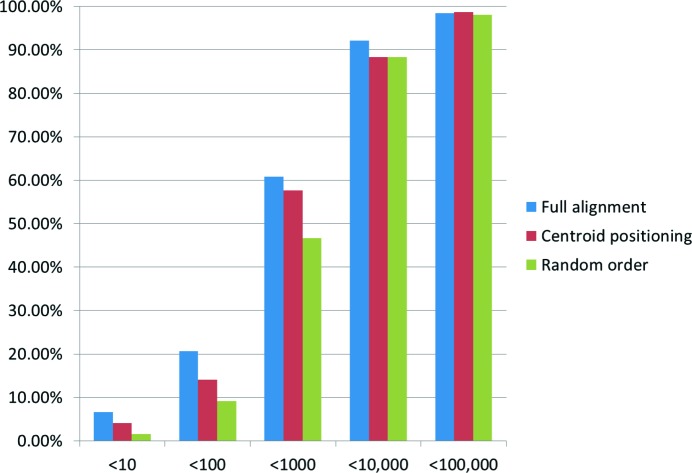
Cumulative number of structural analogues required to retrieve the locally minimized structure for *Z*′′ = 1 structures using alternative approaches. The *x* axis shows the number of structural analogues sampled before the observed minimized structure was generated. The *y* axis shows the percentage of test set entries that were found within the specified number of structural analogues.

The success rate for higher perturbation levels (*i.e.* above 50%), while small, is still significant when considering the efficacy of our method, at least in common space groups and indeed explains why the random selection of structures performs as well as full alignment for higher numbers of considered analogues (Fig. 6 ▸). This suggests that in sampling 100 000 CSD structures our method has many opportunities to optimize to the observed structure from relatively disparate start points.

### 3.2. The benefit of shape and alignment   

Fig. 6 ▸ shows results for a shape-match ordered selection of structural analogues with full alignment, shape match ordered selection of structural analogues with alignments of centroids only and for the randomly ordered selection of structural analogues with full alignment. Both shape-ordered distributions outperform randomly selected entries in the CSD; more good matches are achieved earlier in the search. Applying full alignment between the target molecule(s) and the shape-matched molecule(s) in the structural analogue further reduces the number of structural analogues that must be sampled before the observed structure is retrieved.

Shape-match searching has a noticeable effect on early retrieval of an appropriate structural analogue; both the full alignment and centroid only protocols outperform random ordering, with the full alignment protocol being more than twice as effective as random in the early retrieval of observed crystal structures. Full alignment is of significant benefit within the first 1000 structures sampled. Within 10 000 structural analogues, however, the net benefit of the alignment-based protocols over random selection is relatively small.

Even randomly selected structures retrieve 45% of structures within 1000 trials. One possible explanation for this is that there are certain structures that are members of a large family of entries in the CSD which act as reasonable structural analogues for certain structures. Previous studies (Pidcock & Motherwell, 2004*a* ▸,*b* ▸; Pidcock, 2006 ▸; Braun & Huttner, 2005 ▸) have noted that there are ‘common’ packing arrangements observed in structures within the CSD, which would lend some support to this assertion, suggesting that a subset of the unique packing arrangements of structures in the CSD would reduce the number of analogues required.

12 structures (out of a possible 29 in the random trial) are successfully predicted within 100 analogues selected in both the shape-directed trial and the random trial. Unsurprisingly (given the small probability of choosing the same structure by chance out of 800 000 within 100 attempts), these structures were not generated *via* the same structural analogue in the respective runs of our method. 119 structures (out of a possible 149 in the random trial) are successfully predicted within 1000 analogues selected in both the shape-directed trial and the random trial. This indicates that some structures are easier to predict, as a random selection can yield an analogue structure that is good enough within 1000 random trials.

The space groups of the 119 structures that are predicted in both random and shape-based trials are amongst the more common space groups (*P*1: 1 structure; 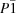: 21 structures; *P*2_1_: 19 structures; *P*2_1_/*c*: 39 structures; *P*2_1_2_1_2_1_: 37 structures and *Pbca*: 2 structures; https://www.ccdc.cam.ac.uk/support-and-resources/ccdcresources/2015_stats_sgrankorder.pdf). Interestingly, the relative rates differ slightly from those generally observed for the CSD, with an enrichment of orthorhombic systems.

We further analysed how often we were able to predict the test set crystal structures by running our method for the first 1000 shape-selected analogues. The number of structures in the test set where multiple analogues could have yielded the observed crystal structure was determined. This showed that for 65% of structures that were predicted successfully, more than one analogue yielded a correct structure within the first 1000 analogues attempted. For 15% of structures that were predicted successfully, more than 5 different analogues yielded the same correct structure, reinforcing the assertion above that certain structures are easier to predict as there are more good analogues on which to base a prediction.

For the test set structures 9α-fluorocortisol (FPRTOD10: Dupont *et al.*, 1972 ▸) and phenylephrine (PHEPHR: Andersen *et al.*, 1976 ▸) we find that 15 structural analogues yielded the observed crystal structure after local minimization. A substantial number of the test set entries including FPRTOD10 that are predicted correctly by more than one different analogue contain characteristic steroidal-like scaffolds (of the 409 test set entries, 43 contain a steroid-like scaffold). Of these 43, 16 are not predicted, 3 are predicted by only one structural analogue while 24 are predicted by more than one structural analogue. This success rate of 62% is equivalent to the success rate of the overall test set within 1000 structural analogues. One can conclude that the CSD is well populated with steroidal analogues. This, however, does not bias the overall conclusions from the full test set.

### 3.3. Selecting unit cells   

Prediction of unit cells is potentially of direct use in any crystal structure prediction method. Indeed, Pidcock & Motherwell (2004*b* ▸) have noted that cell lengths are relatively predictable from a simple box-based model by combining common structural patterns observed in the CSD with molecular dimensions. The analysis of cell retrieval is easily achieved using reduced cell matching (Gruber, 1973 ▸; Andrews & Bernstein, 1988 ▸; McGill *et al.*, 2014 ▸); which gives a measure of similarity with easy-to-interpret tolerances.

For each test molecule, we looked at the first structural analogue found in the same space group (even if this was not the structural analogue that led to a successful prediction) and compared its unit cell to that in the observed crystal structure. The same comparison was then performed between a random structure selected from the CSD in the same space group as the test structure and the observed crystal structure.

The results are shown in Table 3 ▸. The success at the most exacting tolerance level only represents 4 structures in the test set, all of which are correctly predicted directly from the first structural analogue. The results indicate that using the shape metric increases the chance of finding unit cells closer to the experimentally observed cells, which could be used as start points in other CSP approaches.

#### 3.3.1. Selecting the correct space group   

In addition to analysing unit-cell retrieval rates, we were interested in understanding whether shape can aid in predicting space groups. The analysis is described in more detail in the supporting information (see §S5). The conclusion of this analysis, however, shows that one can only gain a large advantage over random if an analogue structure has a very high degree of shape similarity to the actual crystal structure (> 0.95). While interesting, this alone is unlikely to be of great use in most prediction studies, unless a very close shape analogue is known.

### 3.4. Examples of successful predictions   

As noted, there are many examples where an analogue can be found and used successfully. We give a full list of the pairs in the supporting information, but here we show a number of examples where we see successful prediction and discuss their nature.

#### 3.4.1. Isostructurality in structures that have small chemical changes   

**Figure 7:**
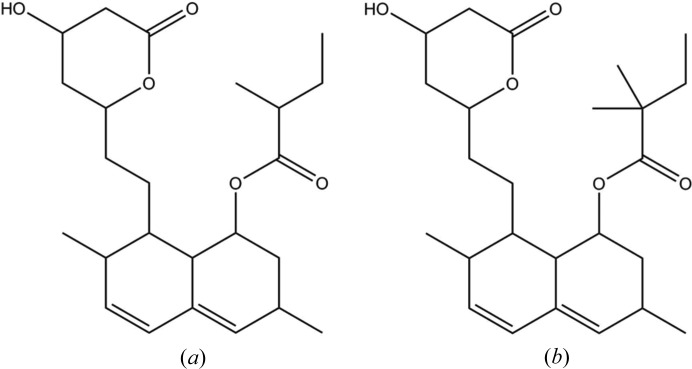
(*a*) Lovastatin (CSD entry CEKBEZ) and (*b*) simvastatin (CSD entry EJEQAL02).

An example of a successful prediction is between lovastatin (CSD entry CEKBEZ: Sato *et al.*, 1984 ▸) and simvastatin (CSD entry EJEQAL02: Hušák *et al.*, 2010 ▸), see Fig. 7 ▸. The structure of these two molecules varies only in the replacement of a H atom with a methyl group. The successful analogue structure (simvastatin) is ranked third by shape.

The second-ranked analogue structure is CSD entry HUDSEE (Kong & Wong, 1999 ▸); this compound is chemically very distinct from lovastatin (a tri-osmium complex). This shows that the reduction of structures into a relatively crude USR shape fingerprint can lead to matches that are not chemically intuitive.

However, another polymorph of simvastatin is the first analogue found by the shape metric (CSD entry EJEQAL01: Hušák *et al.*, 2010 ▸). This would suggest that lovastatin may also form a similar structure. No such structure has been fully characterized as yet; however, thermal characterization of lovastatin has identified crystals with different morphological forms (Yoshida *et al.*, 2011 ▸).

#### 3.4.2. Prediction *via* a chemically related structure   

**Figure 8:**
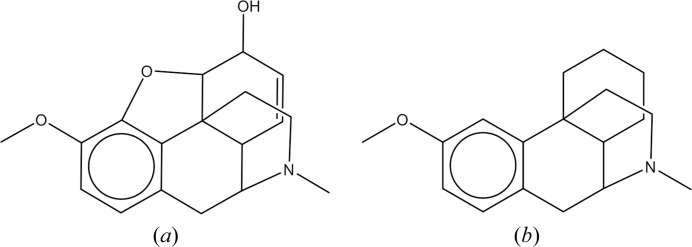
(*a*) Codeine (CSD entry ZZZTSE03) and (*b*) dextromethorphan (CSD entry XAPTAK01); an example of modest isostructurality.

The test set of structures contains the CSD entry for the crystal structure of anhydrous codeine (CSD entry ZZZTSE03: Scheins *et al.*, 2007 ▸). It shape matches to the chemically related molecule dextromethorphan (CSD entry XAPTAK01: Scheins *et al.*, 2007 ▸). Fig. 8 ▸ shows the chemical structures of these two compounds. There is clear similarity between the molecular scaffolds so one would expect a significant degree of shape similarity, XAPTAK01 is the 60th trial structural analogue sampled.

**Figure 9:**
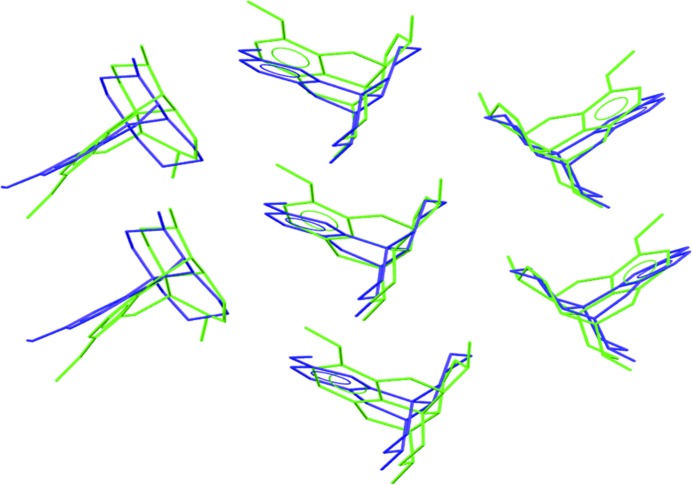
Packing similarity between codeine (in green) and dextromethorphan (in blue). For clarity, a single layer of comparison is shown.

In the crystal structure of codeine, an intramolecular hydrogen bond between the hydroxyl and the ether oxygen is formed rather than an intermolecular hydrogen bond. The codeine molecule adopts the same packing arrangement as dextromethorphan (see Fig. 9 ▸) with an appropriate expansion of the unit cell of the latter to accommodate the additional atoms.

#### 3.4.3. Shape matching without chemical similarity   

**Figure 10:**
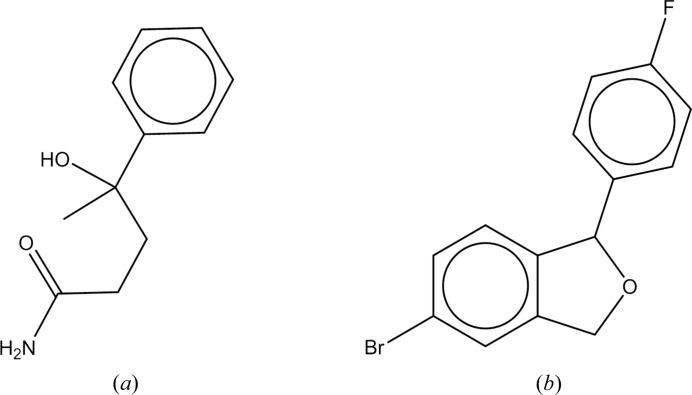
(*a*) γ-Hydroxy-γ-methyl-γ-phenylbutyramide (CSD entry CERNIW) and (*b*) 5-bromo-1-(4-fluorophenyl)-1,3-dihydroisobenzofuran (CSD entry MEHJAL).

Our method relies on the assumption that similarly shaped molecules pack in crystalline lattices in similar ways. For example, γ-hydroxy-γ-methyl-γ-phenylbutyramide (CSD entry CERNIW: Zukerman-Schpector *et al.*, 1984 ▸) and its structural analogue 5-bromo-1-(4-fluorophenyl)-1,3-dihydroisobenzo­furan (CSD entry MEHJAL: Harrison *et al.*, 2006 ▸), which is the 773rd ranked shape match, are structures that have both molecular shape similarity and crystal packing similarity. Chemically, these two molecules are quite distinct (Fig. 10 ▸), but in the crystal lattice both molecules adopt a folded conformation, leading to an overall similarity in shape. In γ-hydroxy-γ-methyl-γ-phenylbutyramide this is due to an intramolecular hydrogen bond between the aliphatic OH and the amide carbonyl which creates a hydrogen-bond mediated seven-membered ring.

**Figure 11:**
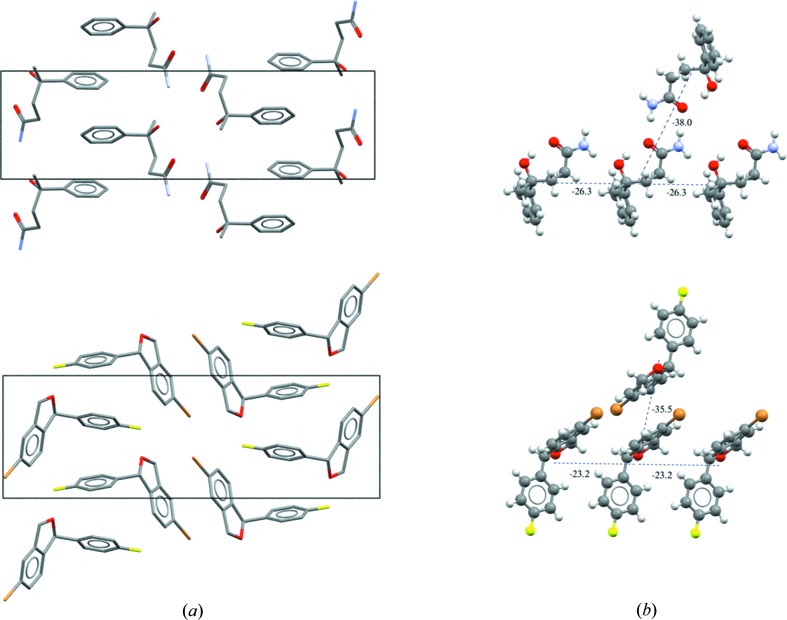
(*a*) A comparison of the relative packing within the respective unit cells of structures CERNIW and MEHJAL. (*b*) A comparison of similar energetic clusters in CERNIW and MEHJAL as predicted by the Unimol force field as implemented in *Mercury* (Macrae *et al.*, 2006 ▸). The dashed lines show the pairwise Unimol interaction energies (kJ mol^−1^).

Analysis of the packing of these two structures helps rationalize the success. Fig. 11 ▸ shows the three strongest interactions between molecules in the respective crystal lattices, and the similar packing arrangement of the two structures.

**Figure 12:**
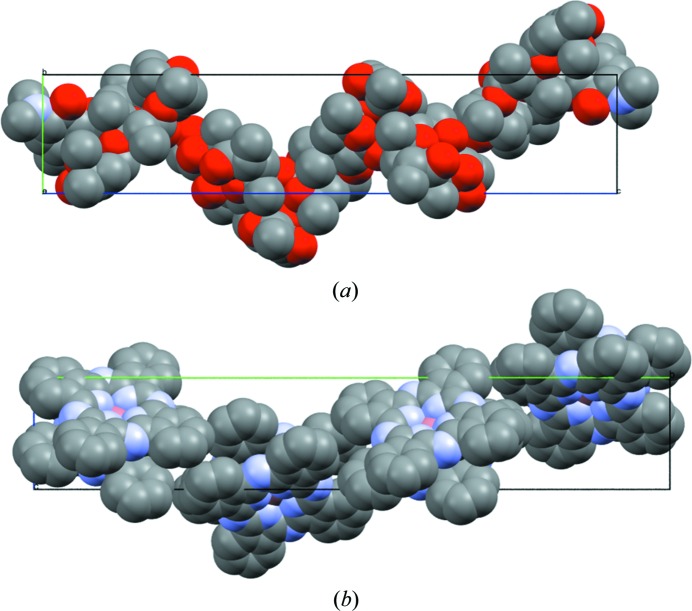
Comparison of molecular packing in structures (*a*) CSD entry QIFKEX01 and (*b*) CSD entry ZAKZOA. Structures are shown in space-filling mode to highlight the similarity in the occupation of the unit cell.

Some structures are predicted successfully from analogue molecules with a low degree of shape similarity. An example is the pair of CSD entries QIFKEX01 (Fujii *et al.*, 2013 ▸) and ZAKZOA (Janczak *et al.*, 1995 ▸). Although these molecules are chemically very different, the analogue structure ZAKZOA was ranked the 3128th by shape similarity. As shown in Fig. 12 ▸, the relative packing and unit-cell dimensions are similar. There is a much higher USR shape ranked (51st) structure (VATFIF: Adachi *et al.*, 1989 ▸), which is also chemically very similar to QIFKEX01, yet yields an alternative minimized structure.

#### 3.4.4. Multi-component systems (*Z*′′ > 1)   

**Figure 13:**
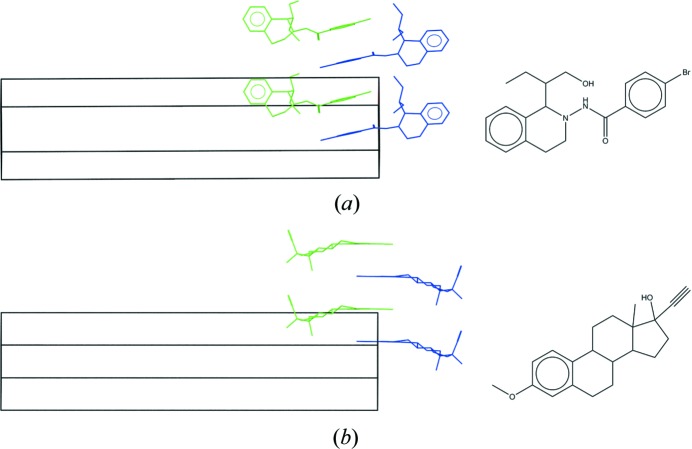
Non-crystallographic symmetry in (*a*) QIXHUC and (*b*) REDNIX. The crystallographically independent molecules are coloured blue and green, respectively. The unit cell is shown in black.

The approach used for prediction in this work is less successful for systems with more than one molecule in the asymmetric unit (*Z*′′ > 1). There were, however, many successful predictions. For example, CSD entry QIXHUC (Li *et al.*, 2014 ▸) was successfully used as a shape analogue for CSD entry REDNIX (Steiner *et al.*, 1997 ▸). Both are *Z*′ = 2, *Z^r^* = 1 structures in the space group *P*2_1_ containing elongated molecules forming dimers between the crystallographically independent molecules that are related to one another by approximate 2_1_ screw axes. The dimers of independent molecules in both cases then align with the longest cell axis (see Fig. 13 ▸).

The presence of approximate non-crystallographic symmetry in this case suggests a possible strategy that may be useful for predicting higher *Z*′ structures. It may be possible to use higher-symmetry crystal structures reduced into higher-*Z*′, lower-symmetry representations to act as analogues. It has been noted that pseudo-symmetry between the related components is common in higher *Z*′ structures; Kuleshova suggest up to 27% (Kuleshova *et al.*, 2003 ▸) and Gavezzotti that 83% of *Z*′ = 2 structures have pseudo-symmetry (Gavezzotti, 2008 ▸), so such an approach would not be without merit.

Some successes also occur in systems with chemically different molecules in the asymmetric unit (*Z^r^* > 1). For example, CSD entry BAHFAR01 (Yathirajan *et al.*, 2004 ▸), is matched by the analogue in CSD entry PEZCUT (Dastychová *et al.*, 2007 ▸). Both structures are ionic systems with a positively charged organic component and a chloride counterion. In effect, the larger organic components are quite similar in shape (though chemically relatively dissimilar).

### 3.5. Chemical similarity as a guide to structural similarity   

One might assume that highly chemically similar structures would be likely to form crystallographically similar structures (Resnati *et al.*, 2015 ▸). Indeed, one of the examples above (lovastatin and simvastatin) is a case in point, where a chemically similar compound leads to a similar packing arrangement in the crystal lattice.

Matched molecular pair analysis of the CSD (van de Streek & Motherwell, 2005 ▸; Giangreco *et al.*, 2016 ▸) has been used to derive lists of CSD entries where a simple molecular transformation exists between a pair of structures. A previous study of chloro–methyl interchange showed that approximately 30% of chloro–methyl structural pairs were isostructural (Edwards *et al.*, 2001 ▸). Indeed, this Cl → Me transformation shows the highest likelihood of a high degree of structural similarity, and has the highest average common cluster size between structures, whereas OH → Me has the lowest likelihood. This is as one would expect: removing a group that has the potential to form a strong hydrogen bond is likely to disrupt the lattice significantly.

**Figure 14:**
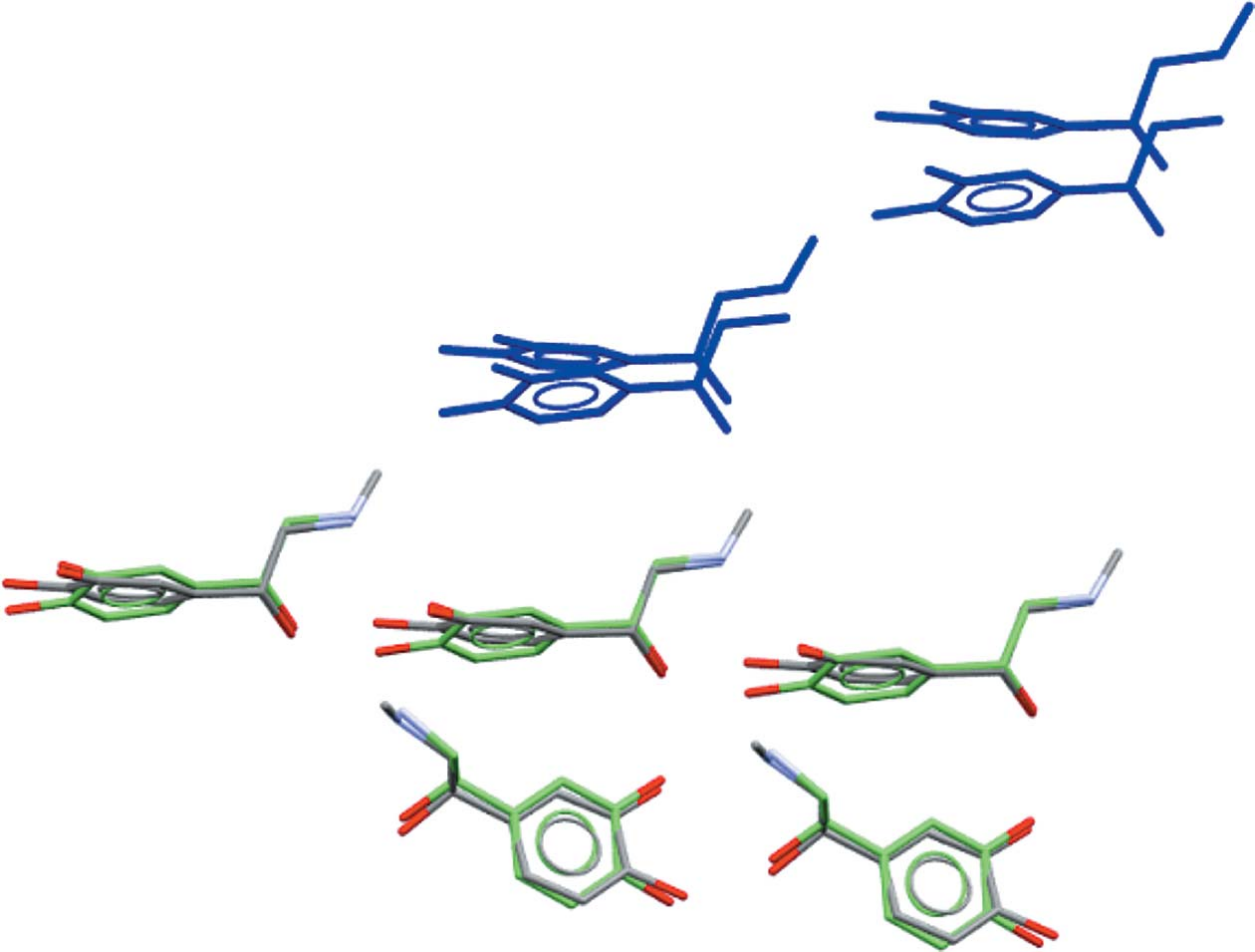
Packing similarity between adrenaline and noradrenaline. The cluster of five molecules in common in the two lattices is shown: noradrenaline has C atoms coloured green, whereas adrenaline is coloured in grey. In blue we see pairs of unmatched molecules in subsequent shells.

A pair of structures where such lattice disruption occurs is adrenaline (CSD entry ADRENL: Andersen, Frandsen *et al.*, 1975 ▸) and noradrenaline (CSD entry NADREN: Andersen, Henriksen *et al.*, 1975 ▸). The transformation of the terminal —NH_3_ in the noradrenaline group into an —NH_2_Me in adrenaline alters the feasible hydrogen-bond patterns available to the respective structures. In noradrenaline, all three available NH donors are used, a pattern that is impossible in adrenaline. The structures do share a significant degree of packing similarity (see Fig. 14 ▸); the additional methyl group forms a weaker CH⋯O contact in place of the missing NH⋯O hydrogen bond. The consequence of the additional methyl, however, is to disrupt the longer range lattice. Adrenaline is an entry in our test set and a shape-matching structural analogue that leads to the observed structure is CSD entry DEVCIR (Kim *et al.*, 2006 ▸); despite the shape similarity it is chemically very distinct from adrenaline.

In certain cases then, chemical similarity could be a useful source of analogue structures to predict crystal structures, but one must consider chemical similarity in the context of the interactions the molecule is likely to make.

### 3.6. Failure case analysis   

We have shown that for the vast majority of single-component (*Z*′ = 1) structures, in the most common space groups, a packing analogue can be found within the CSD. No failures were observed for *Z*′ = 1 structures in the more common space groups (those with more than 10 000 occurrences in the CSD, namely 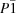, *P*2_1_, *P*2_1_/*c*, *C*2/*c*, *P*2_1_2_1_2_1_ and *Pbca*).

For multi-component systems this is not the case. For 69% of these (we analysed 90 examples) our simple approach to describing shape similarity fails to find a matching structure which packs in the same manner. A significant proportion of these failures (19 out of 67 failing structures) crystallize in the space group 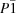, indeed only one multi-component test set entry in 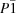 is successful.

Our approach fails for *P*1 and 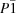 structures more frequently than *P*2_1_/*c* (*P*2_1_/*c* is successful in 96% of cases compared with 64% in 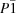 and *P*1 combined); however, structures in these space groups more frequently contain multiple entities in the asymmetric unit. Successful matches can be found for all 33 test cases that crystallize in the space group 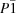 with *Z*′′ = 1. The failure rate observed in the common space group 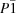 is therefore likely due to the higher relative occurrence of *Z*′′ > 1 structures in this space group within our test set, and indeed within the CSD itself (Taylor *et al.*, 2015 ▸; Steed & Steed, 2015 ▸; see Fig. S3 of the supporting information).

Other failures for single-component structures predominantly occur in less common space groups. We ascribe these failures to the degree of searching undertaken; the method inherently, and deliberately, biases the space searched towards the more common space groups observed in the CSD.

## 4. Conclusions   

Generating structures based on pre-existing CSD structural analogues is useful for the generation of structural lattices. For a test set of 409 predominantly drug-like molecules, the method is successful in generating an analogue that minimizes to the same local minimum as the observed structure in 83.6% of cases, if the first 100 000 analogues suggested by shape similarity are considered.

The method is most successful when tackling single-component structures (*Z*′′ = 1) though challenges remain in predicting multi-component (*Z*′′ > 1) structures. The method used is a demonstration of the potential of shape-based methods for selecting and biasing structural searching, beyond the common approach of using space-group statistics (Price, 2014 ▸), using more of the information available in the CSD.

There are many alternative and more sophisticated methods of shape analysis or molecular alignment that could be used, and alternative protocols that could be adopted (for example, attempting multiple trials in possible analogues) that would likely help further.

We recognize that there are many issues one would need to tackle to make more general use of such an approach. We have not attempted to address conformational flexibility, or the challenge of rapid and reliable scoring. Predicting multi-component systems and structures with molecules on special positions (*Z*′ < 1) requires further development.

Despite these caveats, this approach shows that molecular shape-based selection of starting structures in a crystal structure prediction method could reduce the computational time of the prediction. Such an approach should improve as the number of available crystal structures on which to base predictions increases in the CSD, and may also be aided by access to pharmaceutically relevant structures in private collections.

Whilst we have applied this approach in the context of crystal structure prediction, the authors are cognisant that this methodology has other potential applications, such as structure determination (or, more precisely, identification) from powder data or electron diffraction.

## Supplementary Material

Additional supporting information. DOI: 10.1107/S2052520616006533/gp5082sup1.pdf

Pairs of test set refcodes and the analogues structures used by the program . DOI: 10.1107/S2052520616006533/gp5082sup2.txt

Set of CSD entry identifiers used as the evaluation set. DOI: 10.1107/S2052520616006533/gp5082sup3.txt

Excel file containing shape match probabilities. DOI: 10.1107/S2052520616006533/gp5082sup4.xlsx

## Figures and Tables

**Figure 5:**
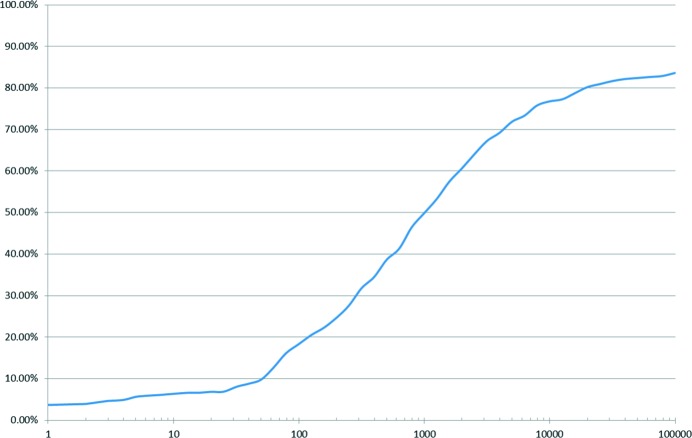
Cumulative percentage of test set entries retrieved within *N* structural analogues for all structures in the test set. The *x* axis shows *N*, the number of structural analogues sampled. The *y* axis shows the percentage of test set entries that were found within the specified number of structural analogues.

**Table 1:** Summary statistics of the 409 test set entries The counts are with respect to the asymmetric unit.

	Minimum	Maximum	Mean	Median
*Z*′	1	4	1.2	1
*Z*′′	1	4	1.3	1
Donor count	0	10	1.9	2
Acceptor count	0	20	5.3	4
Atom count	8	196	46.2	40
Rotatable bond count	0	40	7.49	6
Total atomic weight	44.1	1172.4	353.6	307.4
*R*-factor	0.015	0.211	0.050	0.046

**Table 2:** Breakdown of successes and failures with number of components in the predicted crystal structures

Number of components (*Z*′′)	Number of structures	Successes	Failures
1	319	98% (314)	2% (5)
2	78	33% (26)	67% (52)
3	7	29% (2)	71% (5)
4	5	0% (0)	100% (5)

**Table 3:** Performance of shape matching in predicting unit-cell dimensions The distance tolerance is the maximum allowed percentage difference in cell lengths between the respective reduced cells of the observed structure and the structural analogue. The angular tolerance is the absolute permissible difference in cell angles. The percentage of test set entries retrieved within the respective tolerances are reported.

Distance tolerance (%)	Angular tolerance (°)	First matched analogue in correct space group (%)	Random in correct space group (%)
1.5	2	1.0	0.0
10.0	2	8.5	2.4
20.0	10	27.4	11.1
30.0	15	51.6	24.0
