# Supplementary material for: Generation of crystal structures using known crystal structures as analogues
Source: Acta Crystallogr B Struct Sci Cryst Eng Mater. 2016 Jul 16;72(Pt 4):530–41. doi: 10.1107/S2052520616006533 (PMC4971547; doi:10.1107/S2052520616006533)
Supplement: Supplementary file 1 [file b-72-00530-sup1.pdf]

STRUCTURAL SCIENCE
CRYSTAL ENGINEERING
MATERIALS

Volume 72 (2016)

Supporting information for article:

Generation of crystal structures using known crystal structures as analogues

Jason C. Cole, Colin R. Groom, Murray G. Read, Ilenia Giangreco, Patrick McCabe, Anthony M. Reilly and Gregory P. Shields

S1. Shape match score probability table

The USR shape match algorithm returns shape match values in the range 0 (no match) to 1 (exact match). The generation of candidate crystal structures may involve other forms of scored choice, for example conformer selection scored by log probability. To aid combined scoring, the USR shape match scores were converted to log probabilities by interpolation in a pre-calculated table. The table maps shape match values in 0.01 increments to the log probability of getting or exceeding that shape match value when a molecule, of specified atom count, is shape matched against another molecule of any size.

Shape match value frequency data was collected by shape matching every molecule in 1/20th of the shape database against every molecule in a different 1/20th of the shape database. The log probability tables were calculated from the frequency data. CSD entries in the same family (those that had the same first 6 letters of their CSD refcode) were excluded from comparison. See the associated excel spread sheet for the complete table of log probabilities.

The distribution of shape match probabilities can vary with the number of atoms in the molecules being compared. Normally very high shape match scores (> 0.95) are extremely rare, but for low atom count molecules there is a higher than normal chance of getting a high match score, and high atom count molecules have a higher than normal chance of getting a low match score. There are entries in the table for molecules with between 1 and 20 atoms. Above 20 atoms, the probability distribution was not seen to vary significantly. Figure S1 shows how these probability distributions vary for a number of different atom counts.

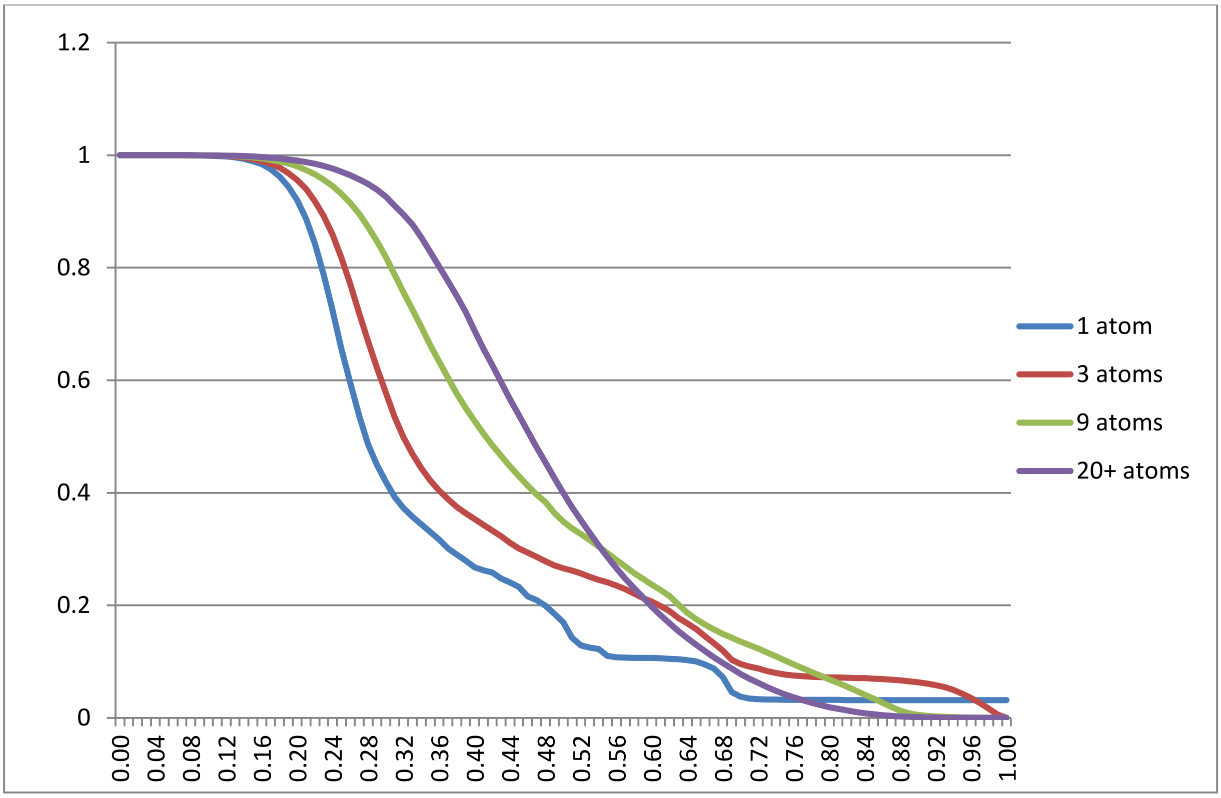

Figure S1 Probability distribution (y axis) of shape match scores (x axis) for various atom counts.

S2. Structure Scoring

S2.1. Inter-molecular score

The inter-molecular score *S(inter)* for a crystal structure is calculated on a per-molecule basis using inter-molecular atom-atom interaction Buckingham potentials:

$$V_B(r) = Ae^{-kr} - \frac{C}{r^6} \qquad (1)$$

To keep the potential finite ranged we calculate the approximate long range r value (r_d) where $V(r_d) = 10^{-3}$ and smoothly switch off V beyond this value (e.g. 11.6 Å for carbon-carbon interactions), over a range of 1 Å so that

$$\begin{aligned} V(r) &= V_B(r)\frac{1}{2}\big(1+\cos\big((r-r_d)\pi\big)\big) && r_d \le r \le r_d + 1 \\ &= 0 && r > r_d + 1 \end{aligned} \qquad (2)$$

To prevent the well-known divergence of $V_B(r)$ at short range, we linearly extrapolate from the point of inflection (r_i) on the repulsive wall, located between the local maximum at short range and the well minimum, where V_B has slope $V_B'(r_i)$ so that

$$V(r) = V_B(r_i) + V_B'(r_i)(r - r_i) \qquad r \le r_i \qquad (3)$$

The parameters *A*, *k* and *C*, based on the Unimol inter-molecular force-field (Filippini & Gavezzotti, 1993 and Gavezzotti & Filippini, 1994), were tuned to improve their ability to replicate CSD crystal structures. The performance of a set of parameters was evaluated by minimising 100-200 CSD crystals that used only those parameters, using a drift index (Gavezzotti, 2011) after minimisation as a badness score. These sets of parameters were then optimised with Limited Memory BFGS (Liu & Nocedal, 1989) to minimise this badness score using numeric gradients.

S3. Effect of refcode family on space group

In the CSD, crystal structure entries are placed into “refcode families” when the underlying molecular structure of the component in the lattice is equivalent. This means that redeterminations of crystal structures at different experimental conditions, polymorphic structures and repeat references to a single structure in the literature are contained within a single family. The reference codes (refcodes) for the distinct entries then differ by a two digit number appended to the reference. For example, structures of the compound sulphanilamide have refcodes SULAMD, SULAMD01, SULAMD03 etc.

Naturally, later codes are often added to the database later in time, when new studies are published. Consequently our conjecture is that later refcodes tend to reflect more extensive studies on compounds than initial studies. In Figure S2, we see the distribution of the space groups in the CSD of the last refcode in refcode families broken down by the count of distinct refcodes in the family for rare and common space groups. The distribution shows a clear trend towards more space group diversity in later family members. One speculative but plausible hypothesis to explain such a distribution is that structures that are later in refcode families are examples of structures which were ‘harder’ to find due to the conditions required for them to form, and were only found when more significant investment was made to discover them.

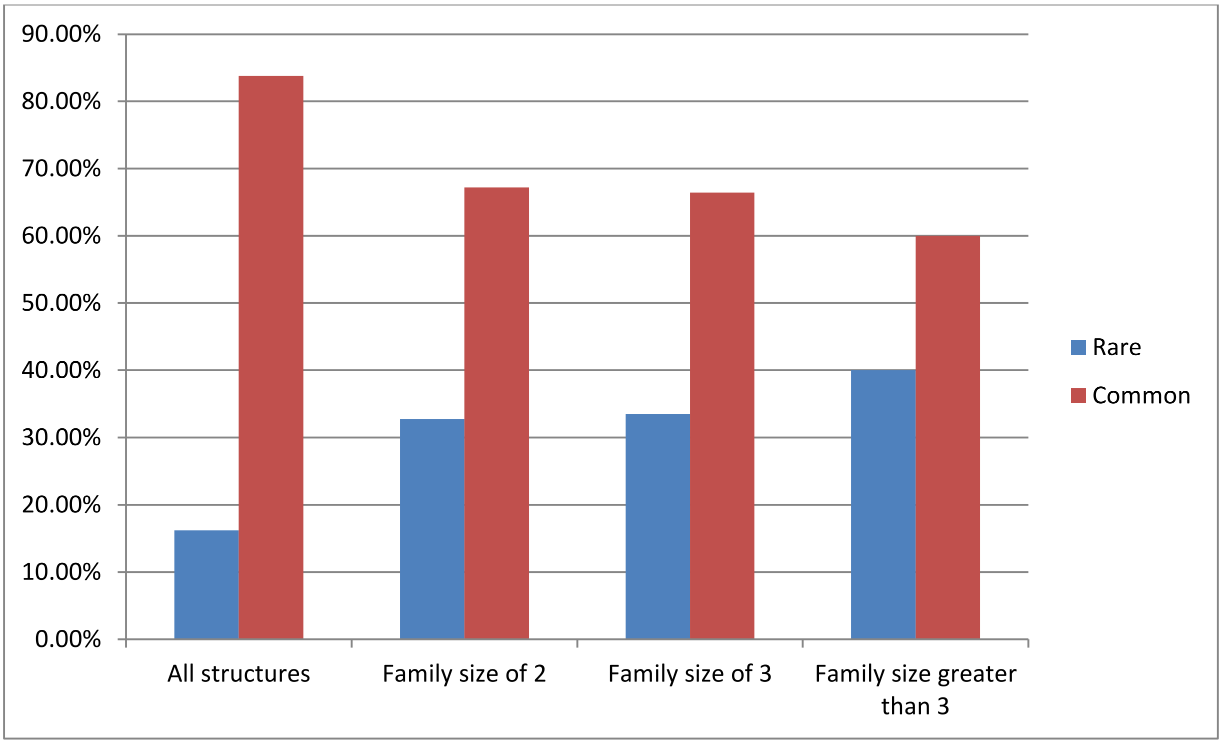

Figure S2 Percentages of rare and common space groups for the last refcode in a refcode family in the CSD broken down by the size of the refcode family. ‘Rare’ structures are defined as those in space groups where there are less than 10000 occurrences in the CSD. ‘Common’ space groups are those where there are more than 10000 occurrences in the CSD.

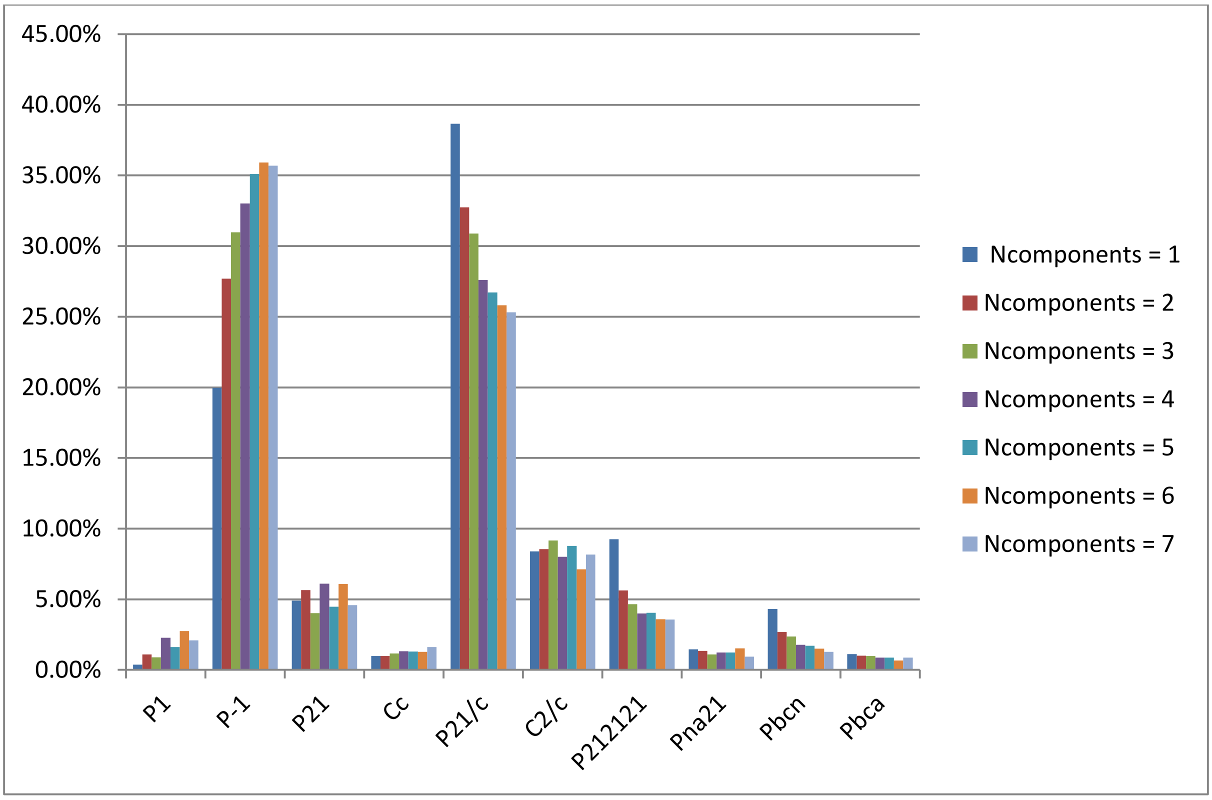

Figure S3 Space group (x axis) against frequency (y axis) split by number of components (Z'')

S4. Response surface in optimisation

We investigated the size of the capture space in local optimization. Each test set structure was perturbed randomly. All variables (rotations, translations and cell parameters were perturbed.). The perturbation applied to each variable was a random amount up to a defined percentage threshold. Following perturbation, the structure was optimised.

The resulting structure was then compared with the observed structure to ascertain if the observed structure was reproduced (structures were considered to be reproduced if the crystal packing similarity (Chisholm & Motherwell, 2005) with a distance tolerance of 20%, an angle tolerance of 20° and a cluster size of 15 molecules gave a (heavy-atom) RMSD less than 0.5 Å.) The process was repeated 10 times for each structure in the test set with each percentage threshold, and the resulting data was used to establish the decline of success with increasing perturbation. The resultant curve is shown in Figure S4.

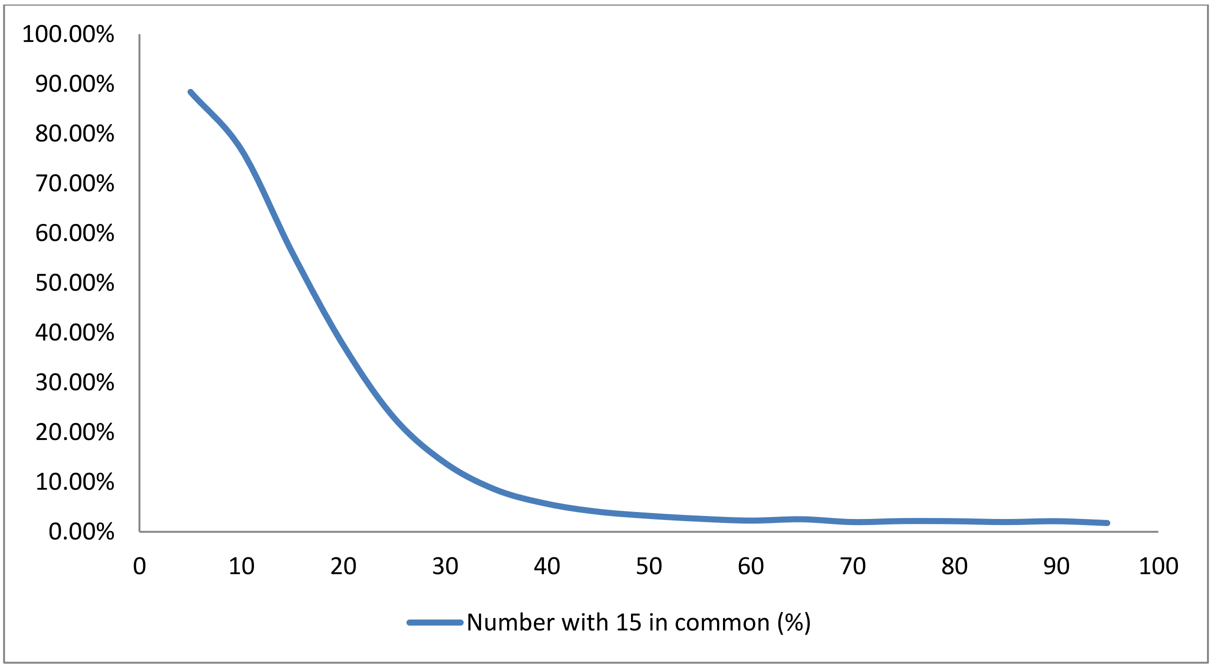

Figure S4 Percentage of local optimisations that lead to the observed structure as a function of starting point perturbation.

S5. Can shape predict space group?

In addition to analysing unit cell retrieval rates, we were interested in understanding whether shape aids in predicting space group. We achieved this by using USR to do a pairwise comparison of all pairs of structures that could be generated from a subset of 133,428 single-component entries in the CSD which were organic, fully determined and had an R-factor < 10.0. This led to 8,901,448,878 comparisons. These comparisons were then sorted by degree of shape similarity, and the percentage of comparisons with the same space group was calculated as a function of shape similarity.

For comparisons with a shape similarity of 0.5, we find that 21% of pairs occur in the same space group. This value can be taken as a baseline: if shape were useful for predicting space group, we would expect higher degrees of shape similarity to lead to higher percentages of pairs in the same space group. As is apparent from Figure S5, there is a slow and gradual increase with similarity, but truly useful enrichment only occurs between very similar structures (with a shape similarity greater than 0.95).

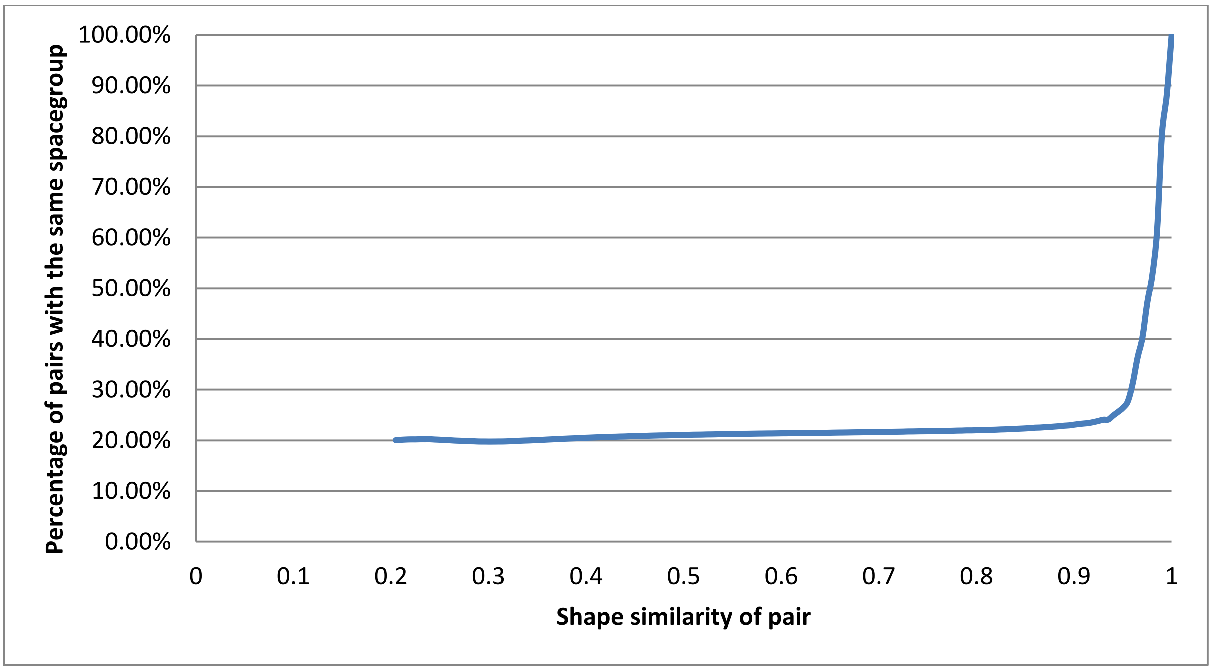

Figure S5 Using shape to predict space group.

S6. Other supplementary files

evaluation_set.gcd : a text file listing the CSD entries used in the test set.

Analogue Pairs.csv : pairs of CSD entries containing the structure predicted and the analogue structure used to predict it.

shape_match_probability_table.xlsx: an excel format spreadsheet containing shape match log probabilities

References

Chisholm, J. A. & Motherwell, W. D. S. (2005). *J. Appl. Cryst.* **38**, 228-231.
Filippini, G. & Gavezzotti, A. (1993). *Acta Cryst.* B**49**, 868-880.
Gavezzotti, A. & Filippini, G. (1994). *J. Phys. Chem.* **98**, 4831-4837.
Gavezzotti, A. (2011). *New J. Chem.* **35**, 1360-1368.
Liu, D. C. & Nocedal, J. (1989). *Math. Program.* **45**, 503-528.
